# Post-Operative Radiotherapy of the Rhabdomyosarcoma R1H of the Rat

**DOI:** 10.1080/13577149778227

**Published:** 1997-12

**Authors:** Ulrich M. Carl, Peter Sminia, Jens Bahnsen, Günter Fröschle, Maria Omniczynski, Lothar Wolf, Uwe Krüger, K. Axel Hartmann, Hans-Peter Beck-Bornholdt

**Affiliations:** 1 Institute of Biophysics and Radiobiology University Hospital Hamburg Germany; 2 University Düsseldorf Clinic for Radiotherapy Moorenstrasse 5 Düsseldorf D- 40001 Germany; 3 Department Radiotherapy AMC University of Amsterdam The Netherlands

## Abstract

*Purpose*. Post-operative radiotherapy (RT) is routinely applied in the treatment of several human tumours. The aim of the present study was to investigate the value of post-operative RT in a rat model.

*Methods*. Experiments were performed using the rhabdomyosarcoma R1H of the WAG/Rij rat. Animals were randomized to different treatment schedules: surgery, RT or a combination of both. Tumours were excised at different sizes (0.1–4.5 g) aiming for complete macroscopic resection. RT (60 Gy in 30 daily fractions over 6 weeks) was applied either primarily or to the former turnout site from the third post-operative day. Tumour growth delay, time to recurrence and local tumour control were used as endpoints.

*Results*. Pre-operative tumour size determined the time and rate of recurrence. The larger the tumour, the shorter the time to relapse and the higher the recurrence rate. The 50% local control rate (LCR_50_) for surgery was found in tumours with a mass of 0.8 g. For post-operative RT a LCR_50_ was achieved for tumours with a mass of 1.1 g. For larger turnouts (> 1.1 g), however, the rate and time course of relapse were similar for both the group receiving RT alone and the group receiving post-operative RT.

*Discussion*. In this model the tumour mass at excision governs the prognosis. Relatively small R1H turnouts may recur despite complete macroscopical resection. With regard to the LCR, the outcome for larger tumours is improved with post-operative RT (60 Gy/6 weeks) than compared with surgery alone. The factor is 1.3. Within a certain range of tumour sizes, combined treatment (surgery + RT) can improve the outcome considerably.


*Sarcoma* (1997) 1, 143–147

CARFAX

ORIGINAL ARTICLE

### Post-operative radiotherapy of the rhabdomyosarcoma R1H of the rat

ULRICH M. CARL,[1,2] PETER SMINIA,[3] JENS BAHNSEN,[1] GÜNTER FRÖSCHLE,[1] MARIA OMNICZYNSKI,[1] LOTHAR WOLF,[1] UWE KRÜGER,[1] K. AXEL HARTMANN[2,3] & HANS-PETER BECK-BORNHOLDT[1]

[1]*Institute of Biophysics and Radiobiology, University Hospital, Hamburg, Germany,* [2]*Clinic for Radiotherapy, University Düsseldorf, Germany &* [3]*Department Radiotherapy, AMC, University of Amsterdam, The Netherlands*

**Abstract**

*Purpose.* Post-operative radiotherapy (RT) is routinely applied in the treatment of several human tumours. The aim of the present study was to investigate the value of post-operative RT in a rat model.

*Methods.* Experiments were performed using the rhabdomyosarcoma R1H of the WAG/Rij rat. Animals were randomized to different treatment schedules: surgery, RT or a combination of both. Tumours were excised at different sizes (0.1–4.5 g) aiming for complete macroscopic resection. RT (60 Gy in 30 daily fractions over 6 weeks) was applied either primarily or to the former tumour site from the third post-operative day. Tumour growth delay, time to recurrence and local tumour control were used as endpoints.

*Results.* Pre-operative tumour size determined the time and rate of recurrence. The larger the tumour, the shorter the time to relapse and the higher the recurrence rate. The 50% local control rate ($LCR_{50}$) for surgery was found in tumours with a mass of 0.8 g. For post-operative RT a $LCR_{50}$ was achieved for tumours with a mass of 1.1 g. For larger tumours ($>1.1$ g), however, the rate and time course of relapse were similar for both the group receiving RT alone and the group receiving post-operative RT.

*Discussion.* In this model the tumour mass at excision governs the prognosis. Relatively small R1H tumours may recur despite complete macroscopical resection. With regard to the LCR, the outcome for larger tumours is improved with post-operative RT (60 Gy/6 weeks) than compared with surgery alone. The factor is 1.3. Within a certain range of tumour sizes, combined treatment (surgery + RT) can improve the outcome considerably.

**Key words:** *rhabdomyosarcoma, rat, post-operative radiotherapy.*

### Introduction

Radical surgical intervention for tumour excision is limited in many cases due to the need to maintain the function of the normal organs concerned. However, one drawback of sparing normal tissue is the enhanced risk of tumour recurrence. Radiotherapy (RT) is a very important additional treatment modality and is a generally accepted approach in curative tumour therapy.[1] When applied post-operatively, RT aims to sterilize tumour cells that might possibly, if not definitely, have remained *in situ* at the time of surgical intervention. Of all the cancer patients who undergo surgery, 30–40% are irradiated post-operatively. A number of questions linked with post-operative RT have yet to be answered, such as at which stage and in which situation will patients profit most.[2]

It appears obvious that the recurrence rate increases with increasing tumour size. In spite of this the actual tumour stage is a poor prognostic parameter. Moderate treatment radicalism in so-called 'low risk' patients can theoretically invert the risk situation. While aiming for small improvements in higher stage tumours, many therapists consider results in early cancers to be sufficient, despite the fact that they can still be improved. For stage I and II gynaecological cancers, it has been shown that treatment results can be improved by post-operative RT.[3,4] The same is true for head and neck tumours.[5] Accordingly, even small breast cancers require 50–60 Gy in breast-conserving treatment due to their multi-focal nature.[6,7] For sarcomas, it has been demonstrated that the metastatic rate increases considerably after local recurrences.[8]

Baker *et al.*[9] have performed a study on different experimental tumours. They showed that tumours

Correspondence to: U. M. Carl, University Düsseldorf, Clinic for Radiotherapy, Moorenstrasse 5, D- 40001 Düsseldorf, Germany. Tel: +49 211 8117994; Fax: +49 211 8118051; E-mail: umcarl@uni-duesseldorf.de.

1357-714X/97/030143-05 $9.00 © 1997 Carfax Publishing Ltd tend to grow better in a post-operative situation—this was attributed to the expression of specific growth factors in the operation wound. Thus, theoretically, cells remaining post-operatively represent the risk of a local recurrence, and may further accelerate the course of the disease unless a valid adjuvant treatment such as RT is applied. In addition, it has been shown for breast cancers[10] and sarcomas[11] that local recurrences can have a negative impact on the survival rate. Thus, local control represents a function of prognosis.[12]

The rhabdomyosarcoma R1H of the rat has been found to be suitable for experimental studies of post-operative RT.[13] The present animal study addressed the question of the role of tumour volume at surgery, the recurrence rate as a function of tumour mass at excision and the value of post-operative adjuvant RT.

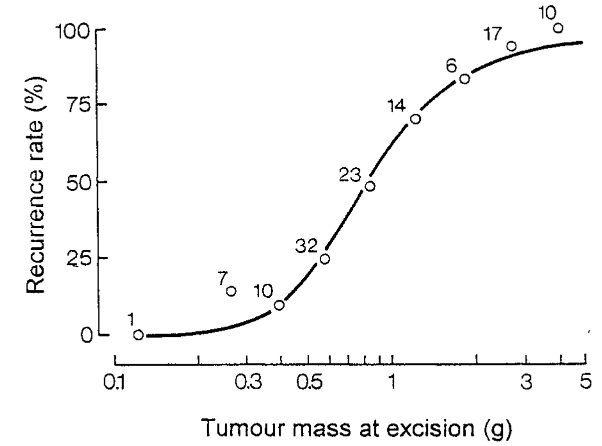

**Fig. 1.** *Tumour recurrence rate of the rhabdomyosarcoma R1H in the rat following surgery as a function of tumour mass at excision. The graph also includes animals that received RT, as accumulated radiation dose early in the course of treatment was unable to prevent temporary recurrence (cf. Fig. 3).*

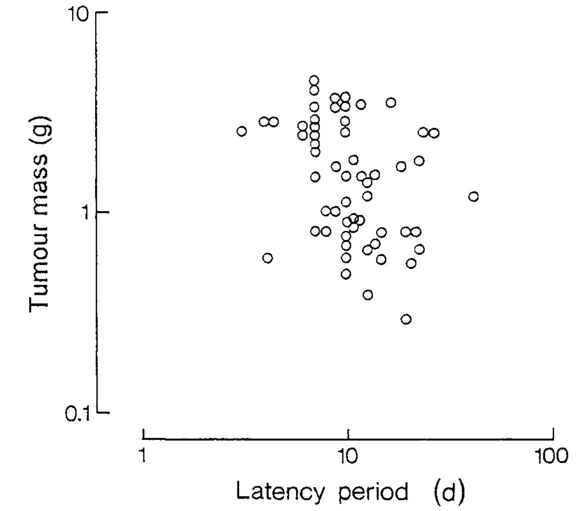

**Fig. 2.** *There is a correlation between the latency period following surgery and the tumour mass. Latency decreases with increasing tumour mass* ($p < 0.001$).

### Materials and methods

Experiments were performed using the rhabdomyosarcoma R1H of the WAG/Rij rat (with no signs of specific immunogenicity).[14] Tumour pieces of about 1 $mm^3$ in size (approx. $10^6$ cells) were transplanted to the right flank of the animals under general anaesthesia. Two to three weeks after implantation the tumours reached treatment size.[13]

A total of 131 animals were randomized to different treatment schedules consisting of RT (control; $n = 8$), surgery ($n = 102$) or a combination of both ($n = 21$). One animal was lost due to intercurrent disease. Control animals were primarily irradiated when their tumours reached the median size of 0.73 $cm^3$ (range 0.6–1.3 $cm^3$). Tumours were excised at different sizes (range 0.1–4.5 g). The extent of excision was limited to the intact pseudocapsule aiming for complete macroscopic resection.[13] None of the animals showed signs of residual tumour masses post-operatively. Local metastatic spread to lymph nodes was not seen.

Tumours and former tumour sites were irradiated locally using 200 kVp X-rays under ambient conditions.[15] RT consisted of five fractions of 2 Gy/week over a period of 6 weeks. Thus, total doses of 60 Gy were applied. In the adjuvant situation, RT was performed from the third post-operative day.

Following treatment, animals were observed three times a week. Size-dependent recurrence rates, as well as specific patterns of treatment response, were subject to further evaluation. Delay in tumour growth was defined as the time to regrow to the start volume $V_0$. To evaluate the mass-dependent recurrence rates, the range of tumour masses was divided into 10 equal intervals on a logarithmic scale (Fig. 1). The actual recurrence rates were then calculated for each intercept and approximated using the maximum likelihood method and assuming Poisson statistics. The non-parametric Spearman's rank correlation coefficient was used to test for a possible correlation between latency time and tumour mass (Fig. 2). Animals that were free of tumour 140 days after the start of treatment were defined to be cured.

The experiments were performed according to the regulations of the veterinarian inspection.

### Results

Surgical curability of the rhabdomyosarcoma R1H of the rat is strongly linked to the tumour mass at excision. After surgery alone, tumours show a 50% local control rate ($LCR_{50}$) at a median mass of

**Table 1.** *Tumour mass as a function of recurrence (R) or cure (C) in tumours receiving 60 Gy post-operatively, also taking into account the clinical observation of temporary recurrences*

| Tumour mass at excision (g) | Temporary recurrence | Final outcome |
|---|---|---|
| 0.65 | Yes | C |
| 0.7 | | C |
| 0.7 | | C |
| 0.7 | Yes | C |
| 0.7 | Yes | C |
| 0.8 | | C |
| 0.8 | Yes | C |
| 0.8 | | C |
| 0.9 | | C |
| 0.9 | Yes | C |
| 0.9 | Yes | C |
| 1.0 | | C |
| 1.1 | | C |
| 1.1 | Yes | R |
| 1.5 | Yes | R |
| 1.5 | Yes | R |
| 2.5 | Yes | R |
| 2.5 | Yes | R |
| 3.0 | Yes | R |
| 3.8 | Yes | R |

There is a significant distinction ($\chi^2$-test: $p < 0.0001$) between the mass of tumours that are finally cured (cf. Fig. 3: ○) and those that recur (cf. Fig 3: ●). Without RT, temporary recurrences would definitely lead to immediate recurrences. This can be avoided ($< 1.1$ g) or postponed ($\geqslant 1.1$ g) by applying 60 Gy post-operatively.

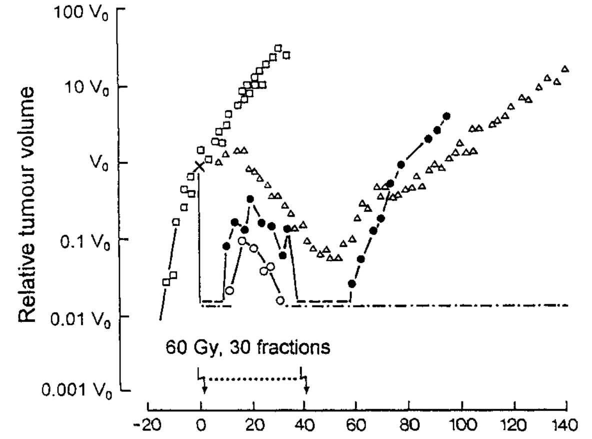

**Fig. 3.** *The relative tumour volume of rhabdomyosarcoma R1H in rats irradiated with 30 fractions of 2 Gy within 6 weeks is shown as a function of time after start of treatment (×). The figure shows the growth curves of the individual tumours with the median response: □, untreated control tumours (historical data, redrawn in parts from Carl[13]); ▼, RT alone (n = 8); ○, local tumour excision (> 1.1 g) and post-operative RT using 60 Gy (n = 12); ●, local tumour excision (≥ 1.1 g) and post-operative RT using 60 Gy (n = 8).*

0.8 g (range 0.7–1.0 g). Below 0.4 g, few recurrences were seen within 140 days of the surgery, but tumours larger than 3.0 g were almost certain to recur (Fig. 1). The graph in Fig. 1 also includes those animals which received post-operative RT (Table 1), as temporary tumours occurred in 13 animals early during RT. Temporary recurrences are defined as tumour recurrences which can be detected shortly after the surgery, and then vanish due to post-operative RT using 60 Gy. In larger tumours ($\geqslant 1.1$ g; $n = 8$) a short period of local control was achieved following 60 Gy, after which tumours recurred. In contrast, smaller tumours ($< 1.1$ g; $n = 12$) were sterilized at the end of RT in spite of temporary recurrences. All recurrences were located within 1.0 cm of the former core of the primary. In most cases the pattern of regrowth was multi-focal. Figure 2 shows that the time to recurrence ranged from 3 to 42 days and decreased with increasing tumour mass ($p < 0.001$).

In Figure 3, median growth curves are presented for the different treatment groups. Tumours receiving 60 Gy irradiation achieved a growth delay of 100 days. Tumours recurred even during post-operative irradiation; however, tumours smaller than 1.1 g could be cured by post-operative RT. Tumours 1.1 g or more vanished beyond the limits of detectability at the end of RT. The median length of this interval of 'complete response' was 3 weeks. For recurring larger tumours ($\geqslant 1.1$ g) that were irradiated post-operatively, the time to regrowth was similar to that for tumours that were irradiated only (Fig. 3).

### Discussion

The present study shows that there is a sigmoid relation between tumour mass at resection and recurrence rate for the experimental rhabdomyosarcoma R1H of the rat (Fig. 1). A preformed tumour bed and the activation of growth factors in the operation wound may have an important impact on the patterns of regrowth, which is faster than after tumour transplantation. The growth kinetics appear to be dependent of the tumour mass at excision (Fig. 2).

Both the present study and former data showed a growth delay of 100 days without any local controls following RT alone using 60 Gy. This was independent of the initial tumour size (Fig. 3).[14] Surgery alone achieved a local control rate of only 50% in tumours with a maximal size of 0.8 g. This figure can be as high as 1.1 g when applying 60 Gy post-operatively. Compared to the results for surgery alone, combined treatment significantly ($p < 0.0001$) pushed up the limits of curability by a factor of 1.3. Large tumours ($\geqslant 1.1$ g range 1.1–3.8 g) recur despite applying 60 Gy after complete macroscopic excision. The latter show a growth delay that is not different from tumours (0.6–1.3 $cm^3$) that have received RT alone (60 Gy). A radiation-induced tumour bed effect can only be seen in the group treated with RT alone. Growth delay not only reflects the effect of irradiation on the tumour cells but also on the stroma, which shows a typical influence on the regrowth pattern following irradiation[16] (cf. Fig. 3).

R1H tumours grow in a pseudo-capsule. Macroscopically, multi-focality is rarely seen in tumours of the size range investigated. Despite complete macrosopical excision the recurrence rate was dependent on the tumour mass. Due to the localization of the recurrences, it does not appear likely that tumour cells are spread in surgery. Moreover, tumour cells outside the pseudo-capsule can cause recurrences in the surgical approach described. Accordingly, tumour cells may be able to cross the capsule and subsequently invade the near vicinity.[17]

The results suggest that, for the R1H tumour system, the extent of remaining tumour formation is a considerable factor in recurrence. Tumour cells migrate eccentrically from the core of a tumour. During the time the primary tumour has to grow, remote cells begin new clones with growth characteristics identical to those of the primary, thus spilling further satellites. Thus, satellites of smaller tumours consist of single cells. This appears likely when one assumes that the spread of satellites is a continuous process, that may progress parallel to, and with the same growth kinetics as, the original tumour. Accordingly, a satellite tumour may develop out of every remote cell. Tumour mass increases with time, during which, satellites may also grow into a state of relative radioresistance. Tumours of 1.1 g have already had enough time to produce satellites that obey similar radiobiological laws as primary tumours. Previously, it was shown that R1H tumours exceeding 1 $mm^3$ cannot be cured by RT alone using 60 Gy.[16] Hypoxia is held responsible for the relatively high radioresistance in the R1H tumour system.[18] Hence, hypoxia might also be important in the satellites of primary tumours larger than 1.1 g.

As the satellites are not yet macroscopically detectable during surgery, they may remain *in situ*. There is no obligatory connection to supplying blood vessels. Accordingly, the actual tumour and its satellites are in a competitive situation. By excising the main tumour, all the nutritive supply of the tumour bed becomes concentrated in the remaining smaller cell formations. This may, in addition to operation-induced growth factors,[9] serve as an explanation for the observation of faster tumour regrowth following surgery rather than after transplantation. The fact that 80% of all recurring tumours occurred within 14 days supports this theory.

The results presented are considered to be of clinical interest. Particularly in early and limited tumours, post-operative RT can sterilize these remaining cells and thus improve outcome. This has been shown for gynaecological cancers,[3,4,19–21] breast cancers,[6,7] and head and neck tumours,[2,5] as well as for sarcomas.[1,12]

Similar to clinical tumours eccentric cell migration plays an important role in experimental tumours. Thus, the migration phenomenon has to be taken into consideration when defining the target volume for RT. Concentric, solid cell formations require larger doses than eccentric single cells, and larger tumours demand larger surgical safety margins. This is despite the need for smaller photon doses at the periphery. On the one hand, penumbra irradiation has a positive impact on the outcome. On the other hand, new and more precise irradiation techniques such as 3-D planned multi-leaf collimator techniques and proton irradiation reduce the size of the penumbra volume, thus increasing the risk of leaving persisting tumour cells untreated.

The experimental data provided show that even small tumours may have an unexpected high recurrence rate following surgery alone, especially when the extent of surgical treatment is limited for several reasons. The outcome can be improved significantly by consequent application of post-operative RT.

### Acknowledgement

Supported by the Spierling-Stiftung, Hamburg, Germany.

### References

1 Schwarz R, Krüll A, Zornig C, *et al.* Strahlentherapie der Weichteilsarkome. In: Roth SL, ed. *Klinische Onkologie 1997, Suppl. Schweizerische Rundschau für Medizin.* 1997:324–32.

2 Naudé J, Dobrowsky W. Postoperative irradiation of laryngeal carcinoma. *Acta Oncologica* 1997; 36:273–7.

3 Carl UM, Bahnsen J, Edel B, *et al.* The value of postoperative radiation therapy in FIGO stage I and II endometrial cancers. *Strahienther Onkol* 1995; 171:322–5.

4 Kucera H, Vavra N, Weghaupt K. Benefit of external radiation in pathologic stage I endometrial carcinoma: A prospective clinical trial of 605 patients who received post-operative vaginal irradiation and additional pelvic irradiation in the presence of unfavourable prognostic factors. *Gynecol Oncol* 1990; 38:618–624.

5 Peters LJ, Goepfert H, Ang KK, *et al.* Evaluation of the dose for post-operative radiation therapy of head and neck cancer: first report of a prospective randomized trial. *Int J Radiat Oncol Biol Phys* 1993; 19:3–11.

6 Fisher B, Constantino J, Redmond C, *et al.* Lumpektomy compared with lumpektomy and radiation therapy for the treatment of intraductal breast cancer. *N Engl J Med* 1993; 328:1581–6.

7 Høst H, Brennhovd IO, Loeb M. Postoperative radiotherapy in breast cancer—long term results from the Oslo study. *Int J Radiat Oncol Biol Phys* 1986; 12:727–32.

8 Cantin J, McNeer GP, Chu FC, *et al.* The problem of local recurrence after treatment of soft tissue sarcoma. *Ann Surg* 1968; 168:47–53.

9 Baker DG, Masterson TM, Pace R, *et al.* The influence of the surgical wound on local tumor recurrence. *Surgery* 1989; 106:525–32.

10 Bahnsen J, Carl UM. Therapeutische Konsequenzen der Früherkennung lokoregionärer Rezidive beim Mainmakarzinom. *Zentralbl Chir* 1993; 118:57–62.

11 Emrich LJ, Ruka W, Driscoll DL, *et al.* The effect of local recurrence on survival time in adult high-grade soft tissue sarcomas. *J Clin Epidemiol* 1989; 42:105–10.

12 Röhrborn A, Röher HD. Chirurgisch-onkologische Aspekte der Sarkomtherapie. In: Roth SL, ed. *Klinische Onkologie 1997, Suppl. Schweizerische Rundschau für Medizin.* 1997:302–12.

13 Carl UM, Beck-Bornholdt H-P, Baumann M, *et al.* Radiotherapy of the rhabdomyosarcoma R1H of the rat: postoperative radiotherapy. *Int J Radiat Oncol Biol Phys* 1990; 18:883–6.

14 Würschmidt F, Beck-Bornholdt H-P, Vogler H. Radiobiology of the rhabdomyosarcoma R1H of the rat: influence of the size of the irradiation field on tumor response, tumour bed effect, and neovascularisation. *Int J Radiat Oncol Biol Phys* 1992; 18:879–82.

15 Vogler H, Beck-Bornholdt H-P. Radiobiology of the rhabdomyosarcoma R1H of the rat: kinetics of cellular inactivation by fractionated irradiation. *Int J Radiat Oncol Biol Phys* 1988; 14:317–25.

16 Beck-Bornholdt H-P, Omniczynsky M, Theis E, *et al.* Influence of treatment time on the response of rat rhabdomyosarcoma R1H to fractionated irradiation. *Acta Oncol* 1991; 30:57–63.

17 Enneking WF, Spanier SS, Malawer MM. The effect of the anatomic setting on the results of surgical procedures for soft parts sarcoma of the thigh. *Cancer* 1981; 47:1005–22.

18 Carl UM. Gemischt fraktionierte Strahlentherapie des R1H Tumors mit Neutronen und Photonen. *Straulenther Onkol* 1993; 10:608–11.

19 Nori D, Hilaris BS, Tome M, *et al.* Combined surgery and radiation in endometrial carcinoma: an analysis of prognostic factors. *Int J Radiat Oncol Biol Phys* 1987; 13:489–97.

20 Pernot M, Hofstetter S, Pfeiffert D, *et al.* Pre-operative, post-operative and exclusive irradiation of endometrial adenocarcinoma. *Strahlenther Onkol* 1994; 170:313–21.

21 Schmitt G, Carl UM, Pape H, *et al.* The role of adjuvant treatment in endometrial cancer. *Strahlenther Onkol* 1994; 170:561–4.
